# A Comment on: An Eocene army ant (2022) by Sosiak CE *et al*.

**DOI:** 10.1098/rsbl.2022.0603

**Published:** 2023-04-26

**Authors:** Dmitry A. Dubovikoff, Dmitry M. Zharkov

**Affiliations:** Department of Applied Ecology, St Petersburg State University, 16th line of Vasilievskiy Island 29, St Petersburg 199178, Russian Federation

A recently published study [1] describes the first finding of a true army ant from the Eocene Baltic amber. The title of the article aroused great interest in the scientific community because true army ants (Dorylinae *sensu* *stricto*) had not been found earlier in the fossil record. The army ants have huge families and are perhaps the most effective predators among ants and other invertebrates. Considering what is commonly called ‘army ant syndrome’, it is curious they have not recovered in the Eastern Hemisphere fossil record. And that is why the article seemed very interesting. However, after a cursory acquaintance with the drawings and the content of the article [1, figure 1*a*], we had doubts about the age and origin of the specimen studied by Sosiak *et al*.

**Figure 1.**
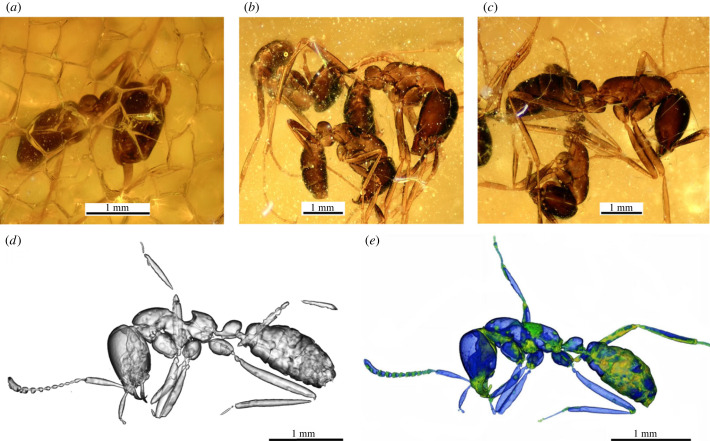
(*a–c*) Specimens of the *Dorylus nigricans* from the African sub-fossilized resin (collection of the Zoological Institute RAS). Micro-CT reconstructions of the specimen PALE-8463, in (*d*) based on a three-dimensional model made by Sosiak *et al.* [2] and (*e*) our results of volumetric rendering of the same specimen. Colours in blue scale are indicated 'void space' and yellow-green tones ‘dense’ areas.

First, the general appearance of the sample [1, figure 1*a*] raised doubts that the authors were dealing with Baltic amber. The nature of the surface (multiple deep and shallow cracks and their position in the form of a fairly dense network) indicate a high probability that this sample is a very young resin (possibly copal) but not Eocene amber. We have at our disposal several dozen similar inclusions of Dorylinae (as well as other insects and ants) from Africa stored at the Zoological Institute of the Russian Academy of Sciences (St Petersburg) that we studied earlier (figure 1*a–c*). The unprocessed (ground and polished) specimens have an identical surface structure (figure 1*a*) to the sample studied by the authors. In addition, the samples studied by us are quite fragile, their surfaces are easily melted during polishing (have a high viscosity) and if briefly exposed to ethanol (5–10 min), their surfaces become sticky. All this highlights the extremely low age of the samples studied. Such a simple test makes it possible to distinguish subfossil resins from amber and helps to avoid such errors in determining the type of fossil resin. The authors failed to do this, but it can be easily tested by them now. Assuming that our conclusions are correct, the sample described by the authors is a young resin and accordingly the results presented in the article are called into question.

In particular, suggested by Sosiak *et al.* [1] in the Introduction, the age of radiation of the main clades of true army ants (EH and WH) is controversial. EH true army ants have still not been found in the fossil record, including, for example, Miocene Ethiopian amber ([3], and our personal data). It is safe to say that the divergence of these clades occurred not before the Oligocene, but actual evidence for this (palaeontological data) has to date not been found.

The next important point on which the authors' conclusions are based is the interpretation of morphological characters of the studied ant. Methods of X-ray microtomography have long been an integral part of the study of fossils, especially in amber. Sosiak *et al.* introduced [1, figure 1*b*] the model they had obtained based on a microtomographic scan of the sample. As the authors themselves note in the caption of the figure [1, figure 1*b*], the model was made on the basis of reconstruction and segmentation of ‘void space’. Indeed, often inclusions in fossil resins are represented by cavities, and ‘void space’ segmentation can be performed easily using most specialized software, just ‘in one click’. However, it must be understood that the results obtained in this way may not reflect the true structure of the specimen and often lead to erroneous interpretation of the data. The model presented by the authors differs from the true morphology of the ant specimen. The structures of the mesosoma (in particular the mesonotum and propodeum) and mandibles, which were one of the main diagnostic features describing the new genus, are not correctly interpreted by the authors. To avoid such errors, the following algorithm should be used. After obtaining an automatic reconstruction of the ‘void space’ and other ‘light’ fractions, the next step should be segmentation (also possible in automatic or semi-automatic mode) of various densities also belonging to our specimen. Now, we can get several segments corresponding to different inclusion densities, which can later be combined into one segment. The next step is the manual segmentation of the missing parts and then the final segmentation during the control of tomographic slices. Next, we can remove inclusions that are not related to our specimen (‘garbage’, air bubbles, etc.). And as a result, we obtain a complete three-dimensional model that we can use to study all the necessary characters of the specimen. By applying the approach mentioned above and based on the initial data by microtomographic scanning, kindly provided by authors [2], we have obtained a full model reflecting the true structure of the described specimen (figures 1*e*, 2) [4,5]. A comparison of the authors' results with the ones we obtained based on their initial data is presented in figure 1*d,e*. Figure 1*e* shows a volumetric rendering of the specimen with a demonstration of the difference in its densities. The blue scale reflects the ‘void space’ segmented by the authors, and the yellow-green tones reflect the missing denser areas. Thus, the data obtained by us fully indicate an incorrect morphological interpretation of the sample. According to our results (figure 2) and electronic supplementary material [4,5], the described specimen does not differ in any way from members of the extant genus *Dorylus* Fabricius, 1793 and the new genus described by the authors should be synonymized with *Dorylus* (=*Dissimulodorylus* **syn. nov.**). As for the described species, based on the features we have studied long scapes and antennae in general, the structure of the mesosoma (promesonotal suture well defined and with deep impression (from lateral view) and the shape of the pygidium), we believe that this species belongs to the *nigricans* group. Summing up, we believe that the army ant described by Sosiak *et al.* [1] as the fossil can actually be attributed to the extant species *Dorylus nigricans* Illiger, 1802 (=*Dissimulodorylus perseus,* **syn. nov.**) encapsulated within the sub-fossilized resin. In the light of the above data, the conclusions of the authors given in the Results and Discussion sections are rendered incorrect and we do not discuss them further.

**Figure 2.**
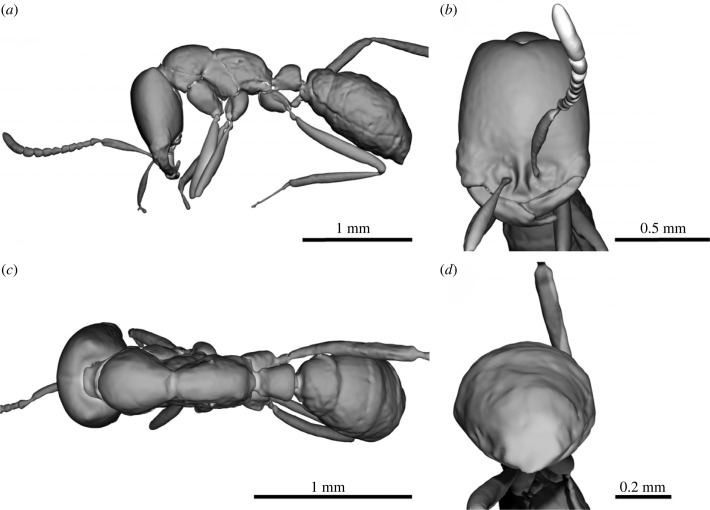
Complete micro-CT reconstructions of the specimen PALE-8463, in (*a*) lateral, (*b*) frontal and (*c*) dorsal view, (*d*) pygidial area.

## Data Availability

Additional materials to support this comment including mCT data (three-dimensional model in format of .stl file) and video file of our model are provided in the electronic supplementary material. The data can be found at the Zenodo Digital Repository: https://doi.org/10.5281/zenodo.7420002 [4] and https://doi.org/10.5281/zenodo.7419984 [5].
